# Emerging trends and hotspots in the links between the bile acids and NAFLD from 2002 to 2022: A bibliometric analysis

**DOI:** 10.1002/edm2.460

**Published:** 2023-11-08

**Authors:** Cong Shibo, Wang Sili, Qiao Yanfang, Gu Shuxiao, Liu Susu, Chai Xinlou, Zhang Yongsheng

**Affiliations:** ^1^ Beijing University of Chinese Medicine, College of Chinese Medicine Beijing China; ^2^ Beijing University of Chinese Medicine, Dongfang Hospital Beijing China

**Keywords:** bibliometric analysis, bile acids, cluster, hotspots, NAFLD

## Abstract

**Background:**

Non‐alcoholic fatty liver disease (NAFLD) is a metabolic syndrome of the liver, and its incidence is increasing worldwide. Accumulating evidence suggests that bile acids are associated with NAFLD. Although many studies on bile acids and NAFLD have been published over the past 20 years, the authors of this study have not found a relevant bibliometric analysis in this field. Therefore, this study aimed to evaluate the trend of publications, summarize current research hotspots and predict future research directions through bibliometric analysis in this field.

**Method:**

Articles related to bile acids and NAFLD published between 2002 and 2022 were obtained from the Science Citation Index‐Expanded of Web of Science Core Collection. Microsoft Excel, CiteSpace, VOSviewer and Bibliometric Online Analysis Platform were used to analyse the publication trends and research hotspots in this field.

**Results:**

Among the articles published between 2002 and 2022, we retrieved 1284 articles related to bile acids and NAFLD, and finally included 568 articles. The USA was dominant until 2020, after which China surpassed the USA to become the dominant force. These two countries cooperate the most closely, and are also the most active in international cooperation. The University of California (UCL) was the most published institution, with a total of 31 publications. There were six authors who have published nine articles and ranked first. The keywords cluster labels show the 10 main clusters: #0fatty liver, #1obeticholic acid, #2oxidative stress, #37 alpha hydroxy 4 cholesten 3 one, #4deoxycholic acid, #5nonalcoholic fatty liver disease, #6mouse model, #7fibroblast growth factor 21, #8animal models, #9high‐fat diet. Keywords burst analysis revealed a higher intensity of study for the nuclear receptor, FXR, and metabolic syndrome.

**Conclusion:**

Bile acids have become an important research direction in the field of NAFLD, and the intervention of gut microbiota in NAFLD by acting on bile acids may become a potential hotspot for future research. This study provides reference and guidance for future research, and will help scholars better explore the field and innovatively discover the mechanisms and treatments of NAFLD.

## Graphical abstract

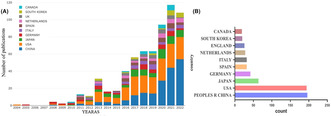

## 1 INTRODUCTION

Non‐alcoholic fatty liver disease (NAFLD) is considered a metabolic syndrome of the liver and is also a progressive liver disease, which mainly includes simple hepatic steatosis, non‐alcoholic steatohepatitis (NASH), liver fibrosis, and eventually cirrhosis.^1^ As a complex metabolic disease, NAFLD is caused by the interaction between genetic and environmental factors and soon became the most common indication for liver transplantation in the United States and Europe.^2^ The major pathological changes of it include simple fatty infiltration, steatosis and hepatic inflammation.^3^ Therefore, it is one of the reasons for the fastest‐growing causes of hepatocellular carcinoma (HCC) incidence worldwide.^4^ Relevant literature predicts that NASH which is an indispensable stage in the course of NAFLD will inevitably become the most common cause of liver cancer in many western countries, with significant clinical relevance to HCC.^5^ The global prevalence of NAFLD is nearly 25%, mainly because it is closely associated with metabolic diseases such as obesity and Type 2 diabetes mellitus (T2D). As the increasing rate of obesity in the global population has changed the normal metabolic status of the organism, how to solve and improve the enormous clinical and economic burden imposed by NAFLD becomes a current problem for governments and medical institutions around the world.^6^ The pathogenesis of NAFLD progresses from simple steatosis or non‐alcoholic fatty liver (NAFL) to NASH, especially NASH combines steatosis with hepatocyte ballooning and inflammation. It may progress to liver fibrosis and eventually lead to cirrhosis and HCC.^2^, ^7^ The disorder of hepatic lipid and lipoprotein metabolism in NAFLD patients not only aggravates the development of NAFLD, but also becomes a major risk factor for the occurrence of cardiovascular disease (CVD) among patients.^8^ Although NAFLD is a pressing global public health problem, the relevant literature on the issue have found that no country is adequately prepared to address it. Therefore, there is an urgent need to develop strategies to address NAFLD at the national and global levels.^9^

Bile acids can be classified into primary, secondary and tertiary, and those synthesized from cholesterol in liver cells are called primary bile acids.^10^ The conversion of cholesterol to bile acids in the liver is the main cholesterol catabolic pathway. Bile acids are secreted into the bile through the apical membrane of hepatocytes, producing bile flow and promoting cholesterol dissolution.^11^ The transformation of cholesterol into bile acids is one of the essential links to maintain cholesterol homeostasis in the body daily, and most bile acids are reabsorbed in the ileum and transported back to the liver via portal blood circulation to inhibit bile acid synthesis.^12^ The enterohepatic circulation of bile acids is an important physiological mechanism for maintaining systemic glucose, lipid, and energy homeostasis to prevent hyperglycemia, dyslipidemia and obesity, as well as preventing inflammatory metabolic diseases in the digestive and cardiovascular systems.^13^ Generally, the effects of bile acids are beneficial to maintain healthy body metabolism, including digesting dietary cholesterol, stimulating intestinal mucosal hormone release, increasing body energy expenditure, reducing liver lipid accumulation and inflammatory response, and reducing endoplasmic reticulum (ER) stress. Relevant Studies have shown that the increase of bile acid content can delay the further development of metabolic diseases.^14^ Bile acids are not only physiological solvents (detergents) required to metabolize dietary fats, steroids and fat‐soluble vitamins, but also signal molecules and endogenous ligands that activate the nuclear farnesoid X receptor (FXR) and G protein‐coupled receptor 5 (TGR5).^15^ Disturbance in bile acid homeostasis can affect the physiological function of normal liver metabolism, which can mainly cause inflammation and promote the pathogenesis of metabolic diseases, such as NAFLD, diabetes, obesity and inflammatory bowel disease (IBDs).^16^ In conclusion, as the scientific studies related to bile acids gradually deepened in recent years, it is found that the association between bile acids and NAFLD is more and more significant, and the number of articles related to the two is also increasing year by year through the search of relevant literature. Therefore, the treatment and delay of NAFLD by regulating the pathways related to bile acid metabolism and maintaining bile acid homeostasis may become a mainstream research focus in the future.

The global prevalence trend of NAFLD and the proliferation of bile acid‐related research hotspots make us urgently study this field and explore its specific mechanism. However, there are few articles summarizing the latest developments in this field and predicting research hotspots. Bibliometric analysis is a timely and comprehensive review of publications in a specific period by analysing the parameters of publications, such as the number of publications, authors, countries and regions, references, keywords, etc. It can provide a detailed overview of the knowledge domains and enable researchers to be made aware of the latest research trends.^17^ At present, there is no global literature on the relationship between bile acids and NAFLD research hotspots and research trends evaluation. The purpose of our study was to analyse the trends of literature publication on the relationship between bile acids and NAFLD from 2002 to 2022, and to explore its potential research hot spots, so as to provide a broader perspective and guidance for other researchers.

## 2 MATERIALS AND METHODS

### 2.1 Data sources and search strategies

**FIGURE 1:**
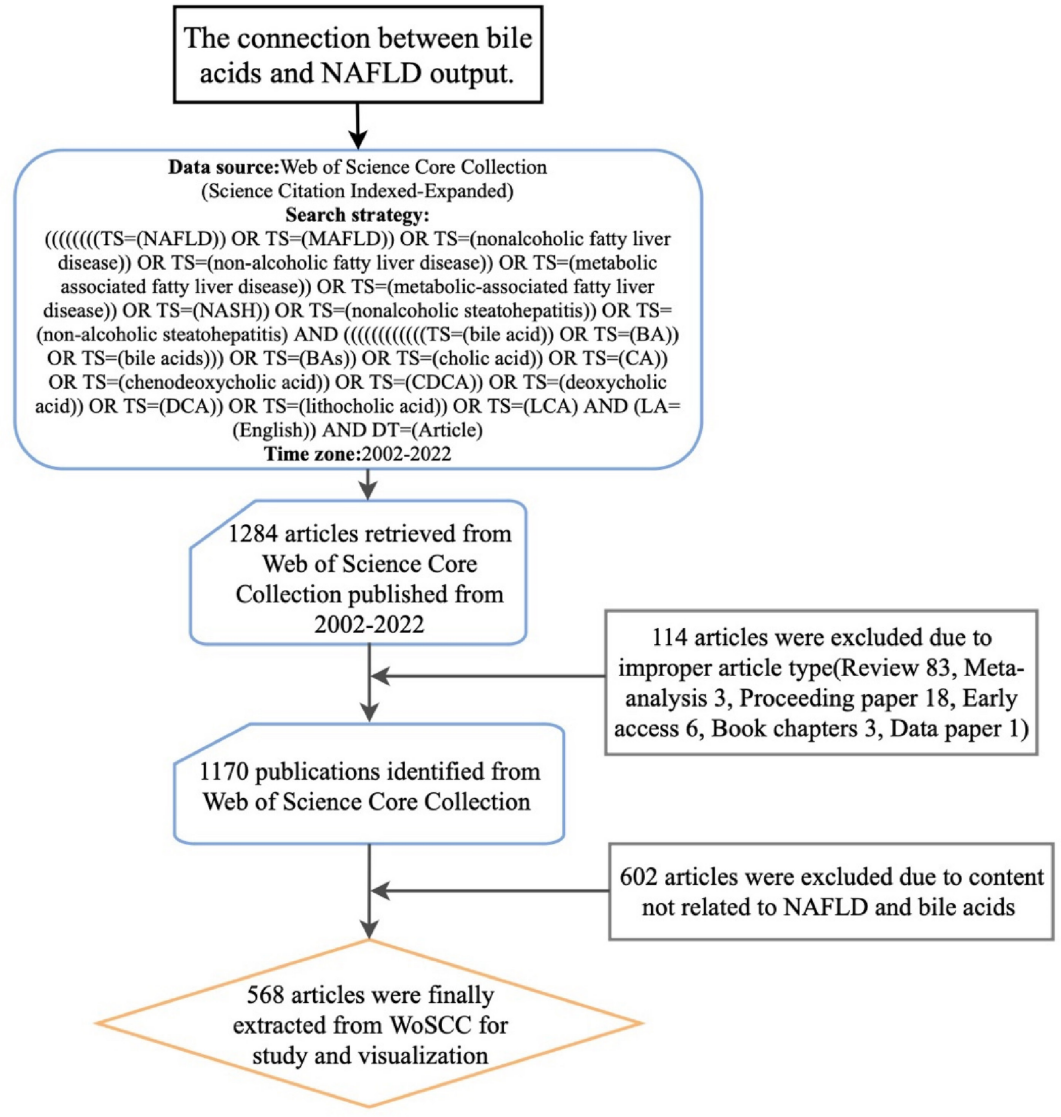
The workflow for retrieving and excluding publications.

We performed a comprehensive literature search of articles published during the period 2002–2022 on 30 March 2023 in the Science Citation Index‐Expanded (SCI‐E) of the Web of Science Core Collection (WoSCC) database. In order to reduce bias caused by frequent database updates, we completed the search and retrieved data within 1 day. Our search strategy was as follows: (#1 AND #2 AND #3); #1 = ((((((((TS = (NAFLD)) OR TS = (MAFLD)) OR TS = (nonalcoholic fatty liver disease)) OR TS = (non‐alcoholic fatty liver disease)) OR TS = (metabolic associated fatty liver disease)) OR TS = (metabolic‐associated fatty liver disease)) OR TS = (NASH)) OR TS = (nonalcoholic steatohepatitis)) OR TS = (non‐alcoholic steatohepatitis); #2 = ((((((((((((TS = (bile acid)) OR TS = (BA)) OR TS = (bile acids))) OR TS = (BAs)) OR TS = (cholic acid)) OR TS = (CA)) OR TS = (chenodeoxycholic acid)) OR TS = (CDCA)) OR TS = (deoxycholic acid)) OR TS = (DCA)) OR TS = (lithocholic acid)) OR TS = (LCA); #3 (LA = (English)) AND DT = (Article). After the initial data retrieval, all manuscripts were screened individually to ensure that all included articles were relevant to the topic of this study and the accuracy of the results of the subsequent bibliometric analysis. The exclusion criteria were as follows: (I) non‐bile acids related, (II) non‐NAFLD related, (III) non‐article type (Proceeding paper, Early access, Book chapters, Data paper, Review and Meta‐analysis) and (IV) duplicate publication. The detailed article screening process is presented in Figure 1. Finally, we included 568 articles on the association between bile acids and NAFLD for bibliometric analysis.

### 2.2 Bibliometric analysis

In order to describe all literature characteristics of articles related to bile acids and NAFLD, we downloaded and saved the data including complete records of the articles and cited references retrieved from Web of Science (https://www.webofscience.com) as TXT format files. And the data files were imported into the Bibliometric online analysis platform (https://bibliometric.com/app), CiteSpace 6.2.R2 software (Drexel University, Philadelphia, PA, USA) and VOSviewer 1.6.19 (Leiden University, Leiden, The Netherlands) for analysis and drawing visualization images, respectively.

The full records and references cited in the publications of the included articles were obtained from the WoSCC database.

And the obtained data were imported into Excel 2019 for analysis and histogram drawing. The number of publications in the top 10 countries/regions and the top 10 most productive journals were analysed and the results were visualized by using a bibliometric online analysis platform. VOSviewer was used to analyse the specific information of the top 10 articles cited. CiteSpace, as a bibliometric software accepted and recognized by most people, was used to analyse the cooperation network of countries and institutions. We also explore core authors, co‐cited articles, keyword bursts, timelines and cluster analysis to help understand the recent trends in bile acid and NAFLD related research and generate potential research hotspots in this field. The generated visual map is composed of nodes and lines, wherein different nodes represent different cited countries, institutions, literatures, authors, keywords and other elements, and the lines between nodes represent co‐occurrence or co‐citation relationships.

## 3 RESULTS

### 3.1 Quantity and trends analysis of published papers

**FIGURE 2:**
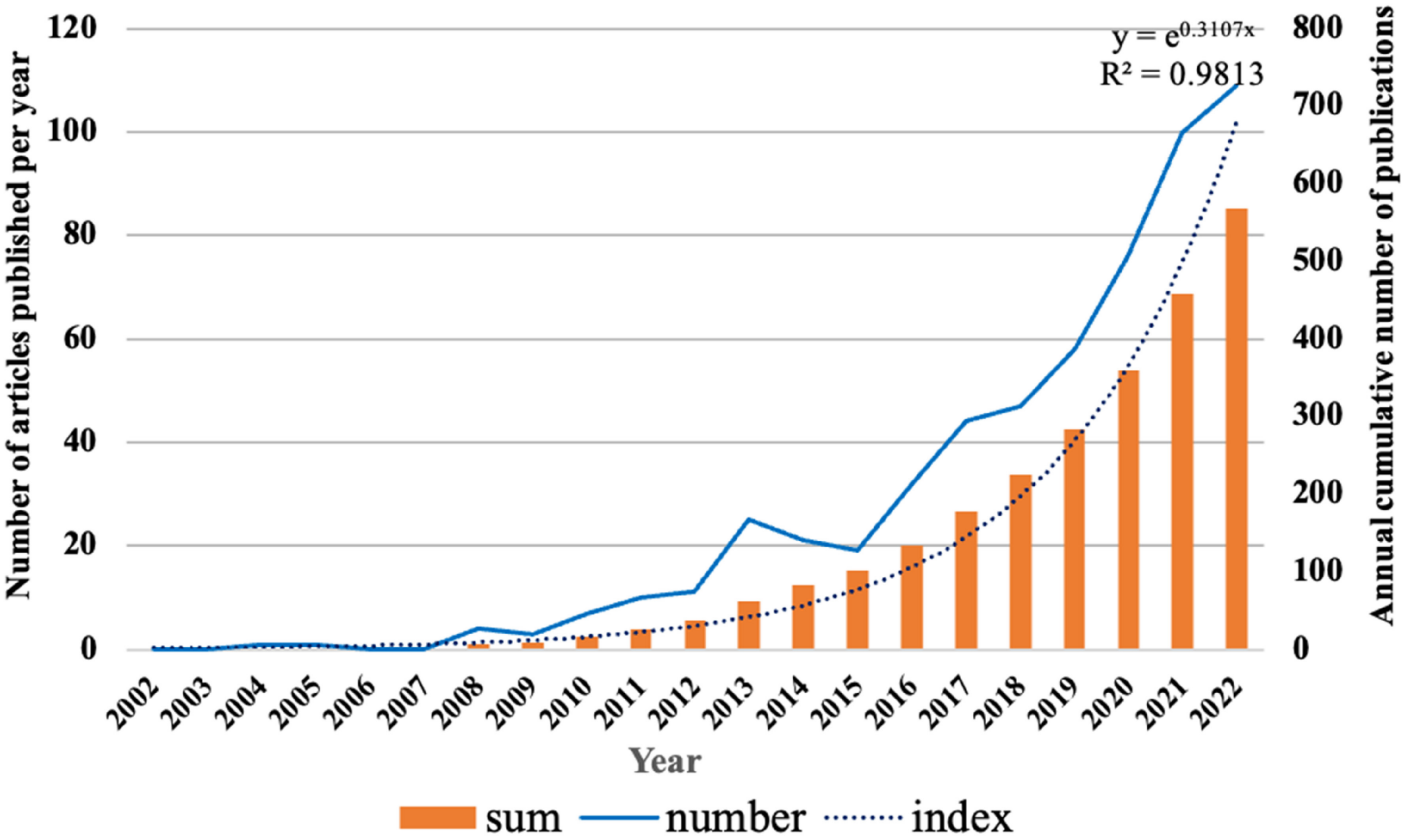
The number of annual and total publications of articles related to bile acids and NAFLD from 2002 to 2022.

In the SCI‐E of WoSCC, the total number of papers published between 2002 and 2022 that met our inclusion criteria was 1284. Seven hundred sixteen articles were excluded due to improper article type and content, including Review 83, Meta‐analysis 3, Proceeding Paper 18, Early access 6, Book chapters 3, Data paper 1, and 602 articles were excluded due to content not related to NAFLD and BA. Based on our search criteria, 568 articles were finally extracted from WoSCC for study. As shown in Figure 2, the research on bile acids and NAFLD can be roughly divided into two periods. The number of articles published in the early stages (2002–2012) grew slowly, while on the contrary the number of articles published began to increase at a higher rate from 2013. The total number of publications in 2013 was twice as much as that in 2012, and the number of publications in 2022 was almost ten times as much as that in 2012. This suggests that the relationship between bile acids and NAFLD as a research hotspot is attracting more and more attention. In addition, we built a growth trend model by using Microsoft Excel 2019 as follows: y = e^0.3107x^ (R^2^ = 0.9813), with a total of nearly 1731 articles predicted to be published until 2025 (Figure 2).

**FIGURE 3:**
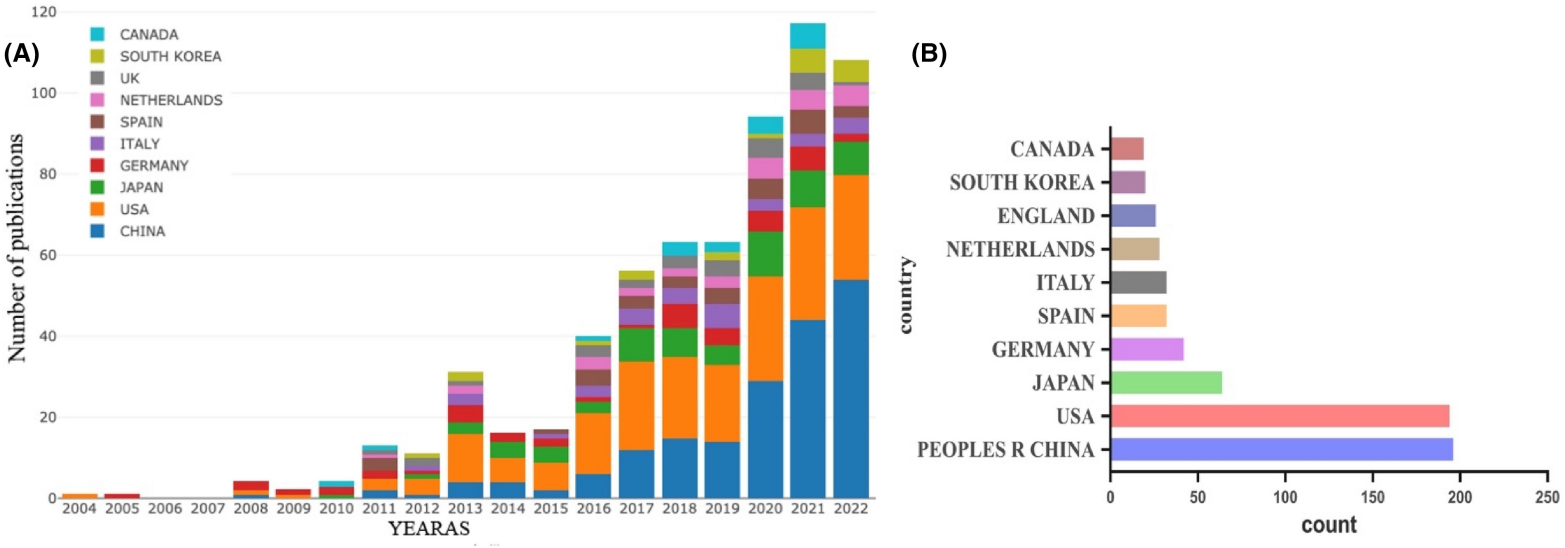
The number of annual publications and growth trends in the field of bile acids and NAFLD from 2004 to 2022. (A) The number of annual publications of top 10 countries in the field of bile acids and NAFLD from 2004 to 2022, export of results from the Bibliometric online analysis platform. (B) The number of total publications of top 10 countries from 2004 to 2022.

In order to understand which country or region has played a leading role in the study of the association between bile acids and NAFLD in the past two decades, the number of articles published in different countries and regions was analysed by Bibliometric online analysis platform (https://bibliometric.com/app). As shown in Figure 3, the bar graph shows the number of publications in the top 10 countries over the 20‐year period. It is worth noting that the United States and China have long dominated the research on bile acids and NAFLD, until 2020, the number of annual publications in China exceeded that in the United States for the first time (China: 29 US: 26) and maintained a growing trend.

### 3.2 Analysis of intercountry/region and interinstitutional co‐operation

**FIGURE 4:**
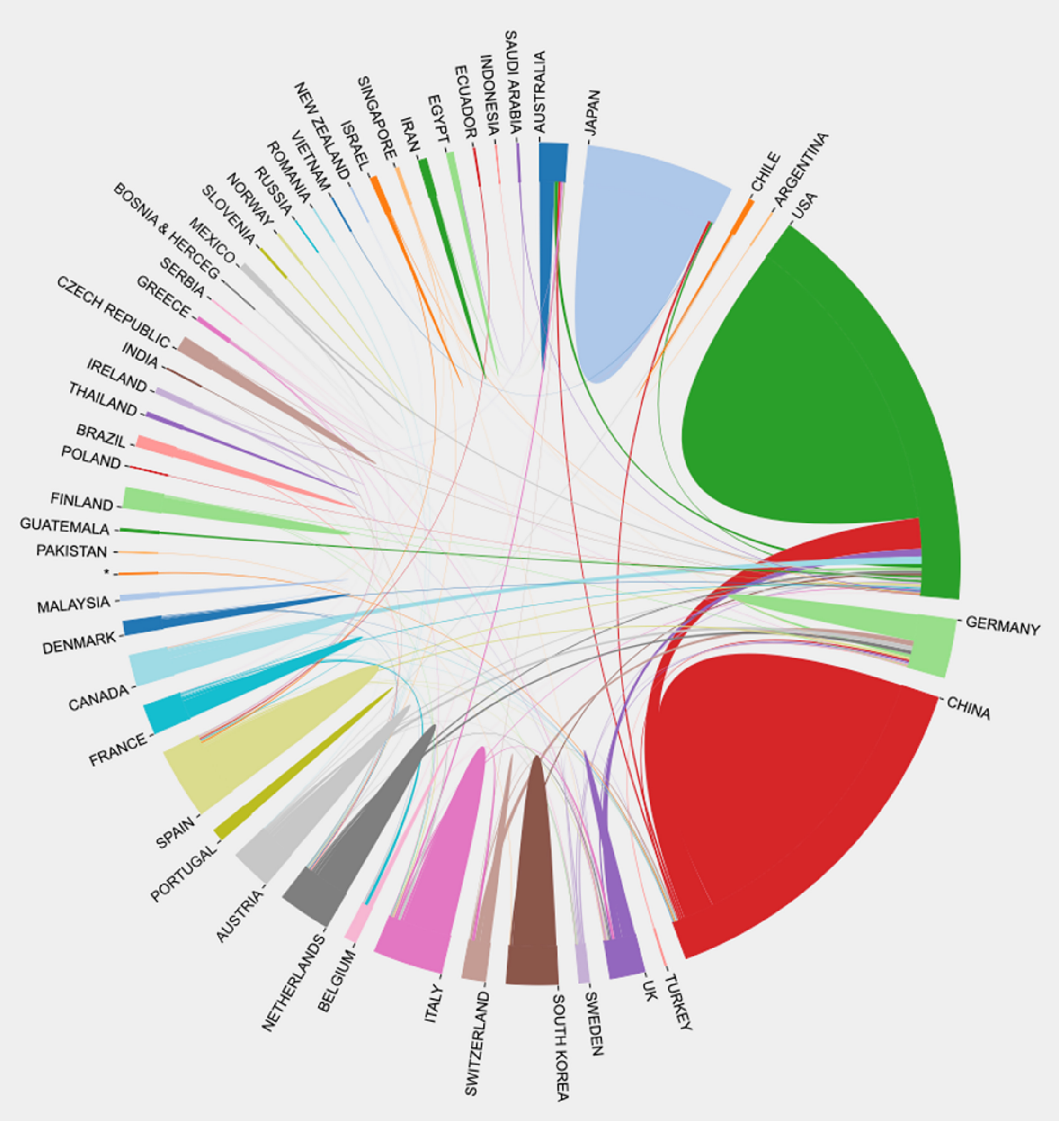
Cooperative relationships between 48 countries in the field of bile acids and NAFLD from 2002 to 2022. The data were exported from the Bibliometric online analysis platform.

A total of 568 articles were published from 48 countries and regions between 2002 and 2022. We used the bibliometric online analysis platform to analyse the cooperation relationship between these countries (Figure 4), and we can find that the United States and China are the two countries that participate in international collaboration most frequently. In addition, these two countries also had the most frequent cooperation between countries/regions, followed by Canada and the United States.

To gain a more intuitive view of the specific number and contribution of publications from each country and institution, we imported TXT format data files into CiteSpace software for analysis. As shown in Table 1, the top 10 countries and institutions in terms of the number of publications are listed in the table. Among them, China had the largest number of publications (196), and its centrality was 0.08. The number of articles published by the United States was only less than that of China, with a total of 194 articles, but its centrality was higher (0.43). Among the top 10 countries in terms of the number of publications, Australia has the highest centrality of 0.76, although it has a small number of articles. However, it is interesting that the density of the network is only 0.0647, reflecting the lack of cooperation between countries, which suggests that we should strengthen academic cooperation and exchanges among different countries in the future.

**TABLE 1:** The top 10 most active countries or regions that published articles in bile acids and NAFLD related research.

Countries	Count	Centrality	Countries	Count	Centrality
PEOPLES R CHINA	196	0.08	USA	194	0.43
JAPAN	64	0.15	GERMANY	42	0.39
SPAIN	32	0.32	ITALY	32	0.15
AUSTRALIA	31	0.76	NETHERLANDS	28	0.35
ENGLAND	26	0.32	SOUTH KOREA	20	0.08

As shown in Table 2, we find that the collaborations between institutions are severely insufficient. Among them, the University of California in the United States has the largest number of publications (31) and the highest centrality (0.19), and has the closest cooperation with other institutions. Fourteen of the 26 institutions in the top 10 in terms of the number of publications (there are multiple institutions with the same number of publications) are from the United States, indicating that American institutions have the largest contribution in this field and play an important role in research. It is known from the above that late development of China in this field, and its publication shows a rapid growth trend after 2020. However, three institutions from China also have a considerable number of publications and rank in the top five list, namely Shanghai University of Traditional Chinese Medicine (18), Shanghai Jiaotong University (15) and Chinese Academy of Medical Sciences (14). In summary, the above analysis also fully demonstrates that institutions from the United States and China play a pivotal role in the research of bile acid and NAFLD.

**TABLE 2:** The top 10 most active institutions that published articles in bile acids and NAFLD related research.

Institution	Count	Centrality	Institution	Count	Centrality
University of California System	31	0.29	Shanghai University of Traditional Chinese Medicine	18	0
Shanghai Jiao Tong University	15	0.03	Chinese Academy of Sciences	14	0.05
National Institutes of Health (NIH)–USA	13	0.06	US Department of Veterans Affairs	13	0.04
University of Groningen	13	0.15	Veterans Health Administration (VHA)	13	0.04
CIBER–Centro de Investigacion Biomedica en Red	12	0.11	University of California San Diego	12	0.24
Cancer Research Center of Hawaii	11	0.02	NIH National Cancer Institute (NCI)	10	0.06
Rutgers State University New Brunswick	10	0.08	Intercept Pharmaceuticals	9	0.01
Medical University of Vienna	9	0.01	University of Hawaii System	9	0.02
University of Kansas	9	0.01	University of Kansas Medical Center	9	0.01
University of Naples Federico II	9	0	CIBEREHD	8	0.1
Harvard University	8	0.04	Institute National de la Sante et de la Recherche Medicale	8	0.12
Medical University of Graz	8	0.15	University of Perugia	8	0
University of Salerno	8	0	University of Texas System	8	0.08

### 3.3 Analysis of co‐authorship network and core author distribution

**FIGURE 5:**
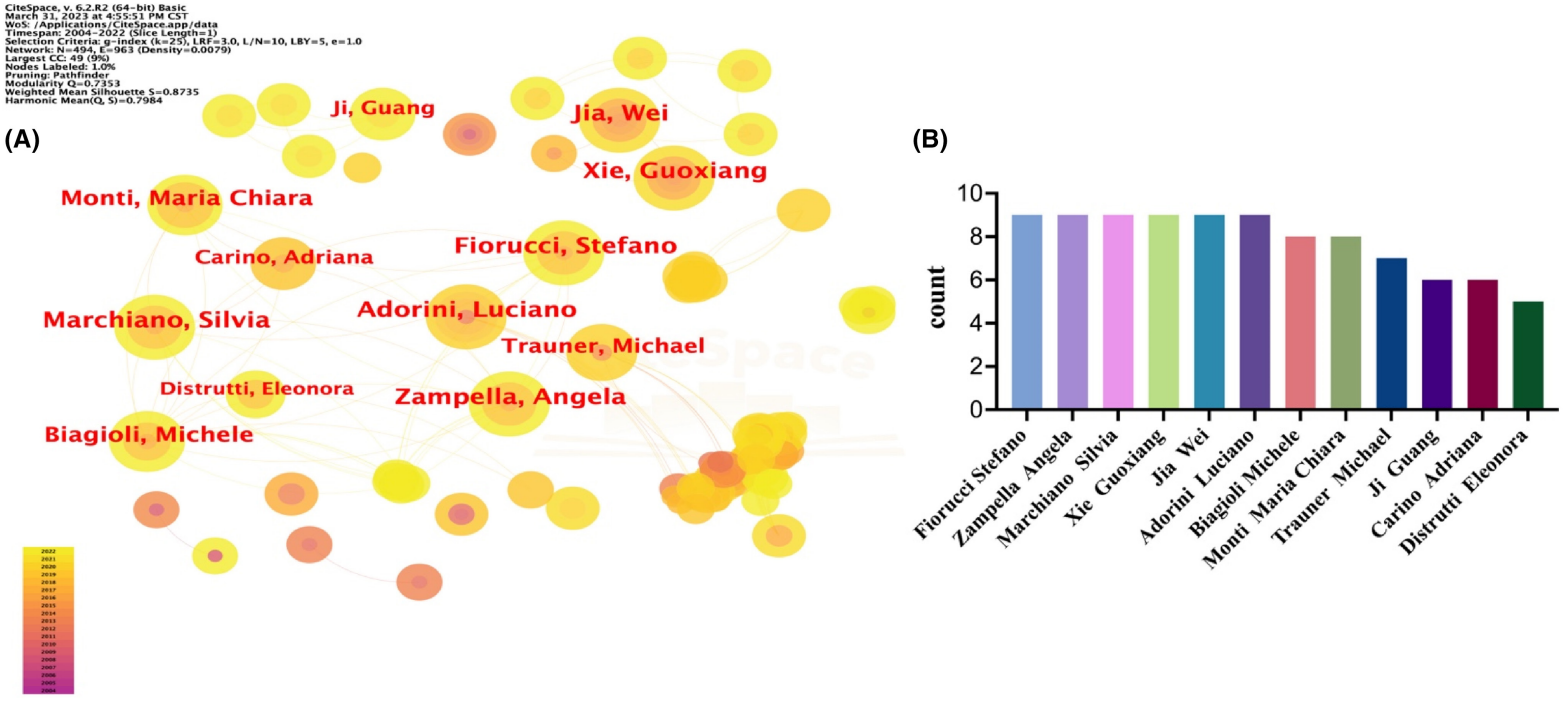
(A) A total of 12 authors with a total number of publications ≥5 in the field of bile acids and NAFLD from 2002 to 2022. Each concentric circle in the figure represents an author, and the line between the concentric circles represents the relationship between the authors. The area of the concentric circle is positively correlated with the number of papers published by the author. The larger proportion of yellow part in the concentric circle indicates that the author has been more active in scientific research activities in this field in recent years. (B) The total number of publications of 12 authors from 2002 to 2022 respectively.

Over the past 20 years, 492 authors have participated in relevant studies of bile acids and NAFLD and contributed to publications. Figure 5 marks a total of 12 authors with a total number of publications ≥5. Each concentric circle in the figure represents an author, and the line between the concentric circles represents the relationship between the authors. The area of the concentric circle is positively correlated with the number of papers published by the author. The larger proportion of yellow part in the concentric circle indicates that the author has been more active in scientific research activities in this field in recent years. Among them, six authors contributed nine publications respectively and ranked first, as shown in Table 3. They are Fiorucci Stefano (University of Perugia, Italy.), Zampella Angela (University of Naples Federico II, Italy.), Marchiano Silvia (University of Washington, USA.), Xie Guoxiang and JiaWei (Shanghai Jiao Tong University, China.), and Adorini Luciano (Intercept Pharmaceuticals, New York, USA.).

**TABLE 3:** The top 10 most active authors that published articles in bile acids and NAFLD related research.

Authors	Count	Centrality	Authors	Count	Centrality
Fiorucci, Stefano	9	0	Zampella, Angela	9	0
Marchiano, Silvia	9	0	Xie, Guoxiang	9	0
Jia, Wei	9	0	Adorini, Luciano	9	0
Biagioli, Michele	8	0	Monti, Maria Chiara	8	0
Trauner, Michael	7	0.01	Ji, Guang	6	0
Carino, Adriana	6	0	Distrutti, Eleonora	5	0

### 3.4 Analysis of journals

Five hundred sixty‐eight articles have been published in 225 academic journals. Bibliometrics online analysis platform was used to analyse the journal influence, Table 4 shows the top 10 journals with the higher number of citations. We found that articles published in *HEPATOLOGY* were cited the most frequently (164), followed by *LANCET* (89), *GUT* (80), *GASTROENTEROLOGY* (68), *JOURNAL OF HEPATOLOGY* (68), *PLOS ONE* (67), *DIGESTIVE DISEASES AND SCIENCES* (63), *EUROPEAN JOURNAL OF GASTROENTEROLOGY & HEPATOLOGY* (62), *METABOLISM‐CLINICAL AND EXPERIMENTAL* (50), *TOXICOLOGY AND APPLIED PHARMACOLOGY* (43), *JOURNAL OF CLINICAL INVESTIGATION* (35). Most notably, *HEPATOLOGY* is also the journal with the largest number of publications, 22 in total. The average number of citations per article published on *LANCET* is up to 89. Eight of the above journals are from the United States, the others from ENGLAND and the NETHERLANDS. According to the latest data (2021), the journal with the highest impact factor (IF) is *LANCET* (IF = 202.731), followed by *GASTROENTEROLOGY* (IF = 33.883), respectively from the UK and the United States.

**TABLE 4:** Top 10 journals with the higher number of citations, the result exported from the Bibliometrics online analysis platform.

Journal	Frequency	Total citations	Average citation per paper	Impact factor (2021)	Country
HEPATOLOGY	22	164	7.45	17.298	USA
LANCET	1	89	89	202.731	ENGLAND
GUT	6	80	13.33	31.795	ENGLAND
GASTROENTEROLOGY	5	68	13.6	33.883	USA
JOURNAL OF HEPATOLOGY	12	68	5.67	30.083	NETHERLANDS
PLOS ONE	18	67	3.72	3.752	USA
DIGESTIVE DISEASES AND SCIENCES	4	63	15.75	3.487	USA
EUROPEAN JOURNAL OF GASTROENTEROLOGY & HEPATOLOGY	2	62	31	2.586	USA
METABOLISM‐CLINICAL AND EXPERIMENTAL	5	50	10	13.934	USA
TOXICOLOGY AND APPLIED PHARMACOLOGY	6	43	7.17	4.46	USA
JOURNAL OF CLINICAL INVESTIGATION	1	35	35	19.456	USA

### 3.5 Analysis of document citations

**FIGURE 6:**
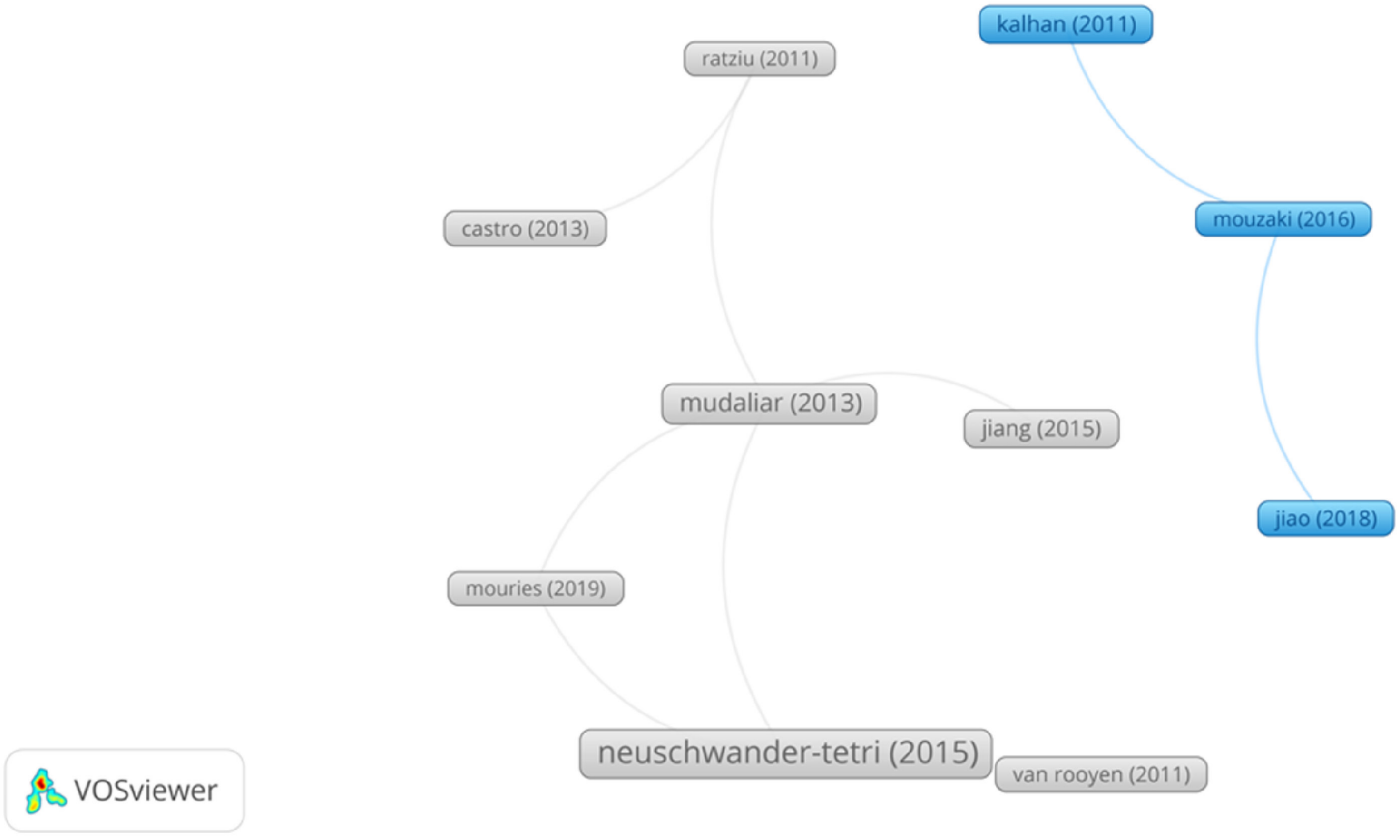
The top 10 articles with the higher number of citations from 568 retrieved articles, result analysed and visualized by VOSviewer.

The number of citations can be used as an important index to evaluate the influence of an article in a certain research field. We also analysed the top 10 articles with the higher number of citations from 568 retrieved articles (Table 5) and visualized them via VOSviewer as shown in Figure 6. In 2015, an article published by Neuschwander‐Tetri et al. from Saint Louis University in the *LANCET* ranked first in terms of citations. Obeticholic acid, a ligand for the Farnesoid X nuclear receptor, was found to improve the histological features of NASH in a multicentre, randomized, placebo‐controlled trial.^18^ The second article published in *GASTROENTEROLOGY* described the efficacy and safety of Farnesoid X receptor agonist obeticholic acid in patients with Type 2 diabetes and NAFLD.^19^ The third is an animal experiment related article published in the *JOURNAL OF CLINICAL INVESTIGATION*. The authors used a high‐fat diet‐induced NAFLD mouse model to determine the impact of gut microbiota alterations on NAFLD, and found that inhibition of intestinal Farnesoid X receptor signalling was identified as a potential treatment for NAFLD.^20^ Of the top 10 most cited articles, three were published in *JOURNAL OF HEPATOLOGY*, followed by two in *GASTROENTEROLOGY*.

**TABLE 5:** Top 10 articles with the higher number of citations, the result exported from the VOSviewer.

Node	Title	First author	Journal	Year	Cited frequency	DOI
**1**	Farnesoid X nuclear receptor ligand obeticholic acid for non‐cirrhotic, non‐alcoholic steatohepatitis (FLINT): a multicentre, randomized, placebo‐controlled trial	Brent A Neuschwander‐Tetri	Lancet	2015	1410	10.1016/S0140‐6736(14)61933‐4.
**2**	Efficacy and safety of the farnesoid X receptor agonist obeticholic acid in patients with type 2 diabetes and nonalcoholic fatty liver disease	Sunder Mudaliar	Gastroenterology	2013	629	10.1053/j.gastro.2013.05.042.
**3**	Intestinal farnesoid X receptor signalling promotes nonalcoholic fatty liver disease	Changtao Jiang	Journal of Clinical Investigation	2015	418	10.1172/JCI76738.
**4**	Plasma metabolomic profile in nonalcoholic fatty liver disease	Satish C Kalhan	Metabolism‐clinical and Experimental	2011	332	10.1016/j.metabol.2010.03.006.
**5**	Suppressed hepatic bile acid signalling despite elevated production of primary and secondary bile acids in NAFLD	Na Jiao	Gut	2018	289	10.1136/gutjnl‐2017‐314,307.
**6**	miR‐34a/SIRT1/p53 is suppressed by ursodeoxycholic acid in the rat liver and activated by disease severity in human non‐alcoholic fatty liver disease	Rui E Castro	Journal of Hepatology	2013	251	10.1016/j.jhep.2012.08.008.
**7**	Hepatic free cholesterol accumulates in obese, diabetic mice and causes nonalcoholic steatohepatitis	Derrick M Van Rooyen	Gastroenterology	2011	223	10.1053/j.gastro.2011.06.040.
**8**	Bile Acids and Dysbiosis in Non‐Alcoholic Fatty Liver Disease	Marialena Mouzaki	Plos One	2016	216	10.1371/journal.pone.0151829.
**9**	Microbiota‐driven gut vascular barrier disruption is a prerequisite for non‐alcoholic steatohepatitis development	Juliette Mouries	Journal of Hepatology	2019	215	10.1016/j.jhep.2019.08.005.
**10**	A randomized controlled trial of high‐dose ursodesoxycholic acid for nonalcoholic steatohepatitis	Vlad Ratziu	Journal of Hepatology	2011	209	10.1016/j.jhep.2010.08.030.

### 3.6 Analysis of document co‐citation and clustered network

Co‐citation is a method of identifying documents cited by a group of authors, this method is used to evaluate the relationship between two articles by visualizing their citation co‐occurrence. Five hundred sixty‐eight articles retrieved from WoSCC and their 17,902 references (excluding self‐citations) were analysed by CiteSpace to determine mutual homogeneity. We listed the top 10 most frequently cited articles in Table 6.

**TABLE 6:** The top 10 most frequently co‐cited articles from 568 retrieved articles, the result exported from CiteSpace.

Node	Title	First author	Journal	Year	Cited frequency	DOI
**1**	Bile acids and nonalcoholic fatty liver disease: Molecular insights and therapeutic perspectives	Arab JP	HEPATOLOGY	2017	64	10.1002/hep.28709
**1**	Suppressed hepatic bile acid signalling despite elevated production of primary and secondary bile acids in NAFLD	Jiao N	GUT	2018	64	10.1136/gutjnl‐2017‐314,307
**2**	The presence and severity of nonalcoholic steatohepatitis is associated with specific changes in circulating bile acids	Gottlieb A	HEPATOLOGY	2018	61	10.1002/hep.29359
**2**	Farnesoid X nuclear receptor ligand obeticholic acid for non‐cirrhotic, non‐alcoholic steatohepatitis (FLINT): a multicentre, randomized, placebo‐controlled trial	Neuschwander‐Tetri BA	LANCET	2015	61	10.1016/S0140‐6736(14)61933‐4
**3**	Global epidemiology of nonalcoholic fatty liver disease‐Meta‐analytic assessment of prevalence, incidence, and outcomes	Younossi ZM	HEPATOLOGY	2016	41	10.1002/hep.28431
**4**	Intestinal Crosstalk between Bile Acids and Microbiota and Its Impact on Host Metabolism	Wahlstrom A	CELL METAB	2016	37	10.1016/j.cmet.2016.05.005
**4**	Bile Acids and Dysbiosis in Non‐Alcoholic Fatty Liver Disease	Mouzaki M	PLOS ONE	2016	37	10.1371/journal.pone.0151829
**5**	Bile Acid Control of Metabolism and Inflammation in Obesity, Type 2 Diabetes, Dyslipidemia, and Nonalcoholic Fatty Liver Disease	Chavez‐Talavera O	GASTROENTEROLOGY	2017	36	10.1053/j.gastro.2017.01.055
**6**	Mechanisms of NAFLD development and therapeutic strategies	Friedman SL	NAT MED	2018	34	10.1038/s41591‐018‐0104‐9
**7**	The diagnosis and management of nonalcoholic fatty liver disease: Practice guidance from the American Association for the Study of Liver Diseases	Chalasani N	HEPATOLOGY	2018	32	10.1002/hep.29367
**8**	Global burden of NAFLD and NASH: trends, predictions, risk factors and prevention	Younossi Z	NAT REV GASTRO HEPAT	2018	30	10.1038/nrgastro.2017.109
**9**	Altered Bile Acid Metabolome in Patients with Nonalcoholic Steatohepatitis	Ferslew BC	DIGEST DIS SCI	2015	27	10.1007/s10620‐015‐3776‐8
**10**	The severity of nonalcoholic fatty liver disease is associated with gut dysbiosis and shift in the metabolic function of the gut microbiota	Boursier J	HEPATOLOGY	2016	23	10.1002/hep.28356
**10**	Efficacy and safety of the farnesoid X receptor agonist obeticholic acid in patients with type 2 diabetes and nonalcoholic fatty liver disease	Mudaliar S	GASTROENTEROLOGY	2013	23	10.1053/j.gastro.2013.05.042

We found that the most cited reference was the 2017 *HEPATOLOGY* journal published with the title ‘Bile acids and nonalcoholic fatty liver disease: Molecular insights and therapeutic perspectives’.^21^ The relationship between bile acids and NAFLD and the possibility of treating NAFLD by targeting bile acid‐related pathways are discussed in detail. In 2018, an article about animal experimental research published in the *GUT* titled ‘Suppressed hepatic bile acid signalling despite elevated production of primary and secondary bile acids in NAFLD’ was also ranked first.^22^ This study suggests that intervention of NAFLD can be achieved by targeting FXR signalling pathways, including bile acids conversion and gut microbiota. The second ranked articles are ‘The presence and severity of nonalcoholic steatohepatitis is associated with specific changes in circulating bile acids’ published in *HEPATOLOGY* in 2018 and ‘Farnesoid X nuclear receptor ligand obeticholic acid for non‐cirrhotic, nonalcoholic steatohepatitis (FLINT): a multicentre, randomised, placebo‐controlled trial’ published in *LANCET* in 2015.^18^, ^23^ These two articles respectively discuss the relationship between the presence and severity of NASH and specific changes in circulating bile acids, as well as the improvement of the histological characteristics of NASH by Farnesoid X nuclear receptor ligand obeticholic acid. The article ranked third was published in *HEPATOLOGY* in 2016, it was about the analysis and evaluation of epidemiological indicators related to NAFLD.^6^ These articles discuss the pathophysiology of NAFLD and potential treatments from clinical studies to the molecular level. It can be considered that the articles with high citations listed in the Table 6 have made great contributions to the research in the field of bile acids‐NAFLD and are the most recognized by most scholars.

### 3.7 Analysis of the research trend and burst detection with keywords

**FIGURE 7:**
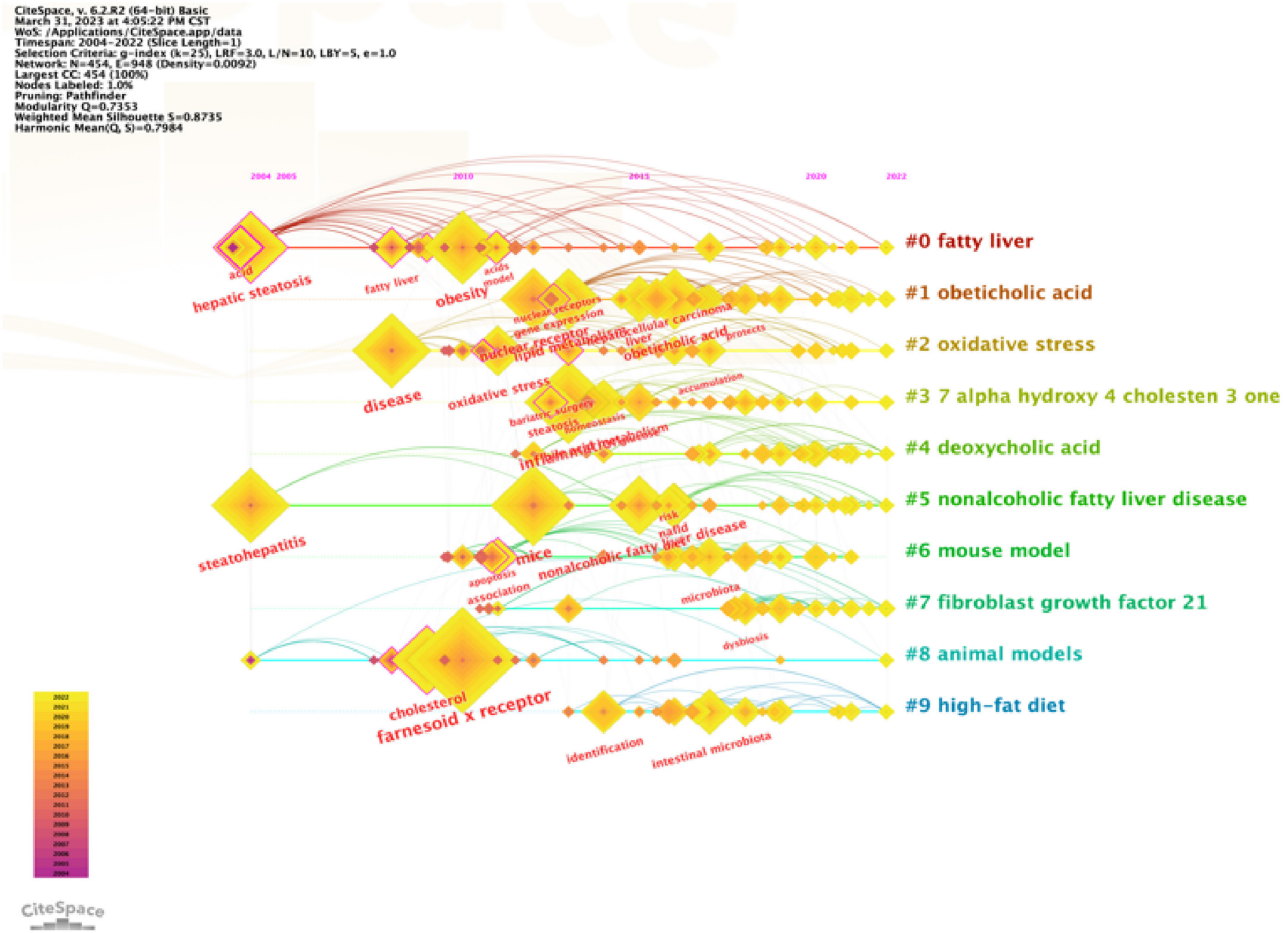
The time‐Line chart of the top 10 largest cluster analysis of keywords in the field of bile acids and NAFLD. Each quadrilateral represents the papers that are mainly cited in a certain cluster, and the rings of different area sizes on the timeline represent the frequency of citations.

In order to clearly describe the evolution of research hotspots on bile acids and NAFLD over the past 20 years, we used CiteSpace to perform a cluster analysis of keywords and visualize them. As shown in Figure 7, each quadrilateral represents the papers that are mainly cited in a certain cluster, and the rings of different area sizes on the timeline represent the frequency of citations. We found that hepatic steatosis, steatohepatitis, FXR receptor and inflammation were the research hotspots in the field of bile acids and NAFLD. Hepatic steatosis began in 2004 and is still a research hotspot until 2022. Since 2010, liver FXR receptors have gradually become a research hotspot and become the most cited cluster by 2022, followed by obesity. Inflammation and nuclear receptors have gradually replaced steatohepatitis as a research hotspot in this field since 2013. In general, the focus of bile acids and NAFLD seems to have shifted from steatosis, inflammation and FXR receptors to bile acids clusters including obeticholic acid and the gut microbiota.

**FIGURE 8:**
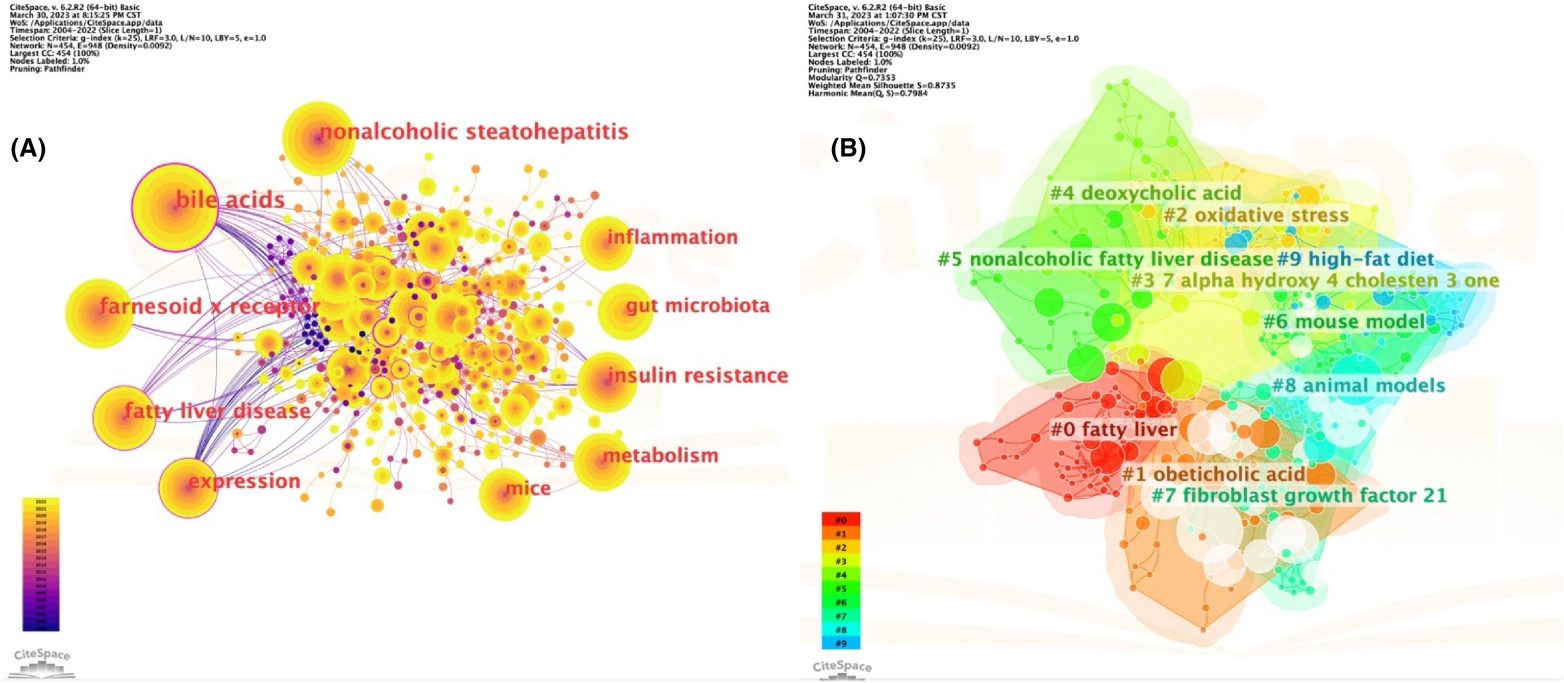
(A)The top 10 largest keywords with higher citations analysed and visualized by CiteSpace. (B) The top 10 largest clusters of citing articles in the field of bile acids and NAFLD research. Clustered networks of keywords analysed and visualized by CiteSpace.

As shown in Figure 8A, we analysed keywords in the field of bile acids and NAFLD research in order to identify the research frontiers in this field well. The top 10 keywords were bile acids (213), nonalcoholic steatohepatitis (156), farnesoid X receptor (137), fatty liver disease (121), expression (108), insulin resistance (107), metabolism (99), gut microbiota (88), inflammation (86) and mice (82). We further conducted a cluster analysis based on the connection strength between keywords and visualized the top 10 cluster labels, as shown in Figure 8B. The labels of the clusters represented the keywords in the cluster were relatively uniform, and the colour area of each cluster was positively correlated with the number of keywords it contained. We found that cluster #1 Obeticholic acid and cluster #2 Oxidative stress, which were the larger group among the 10 clusters.

**FIGURE 9:**
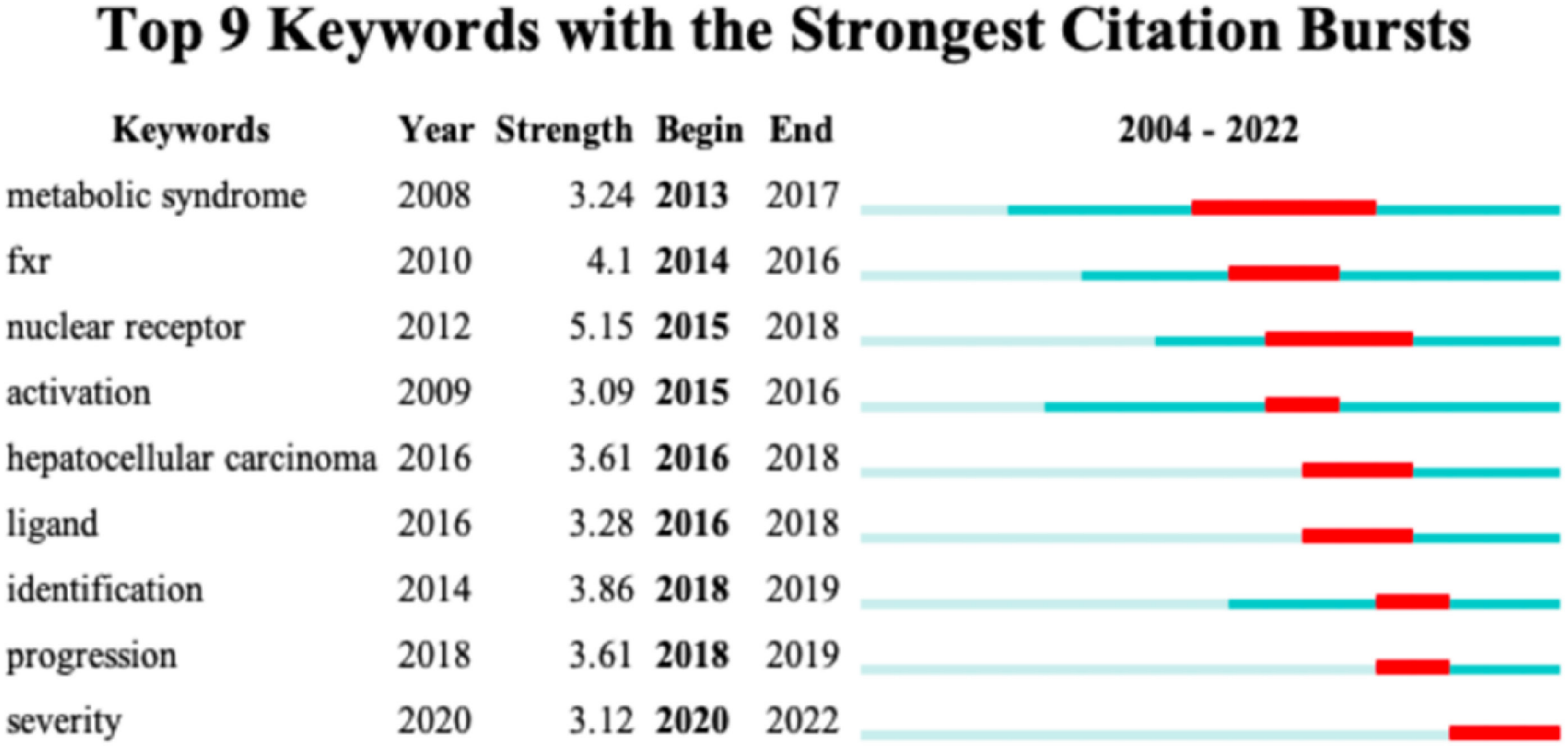
Keywords with the strongest burst strength of the 568 articles on bile acids and NAFLD related research from 2002 and 2022. The blue line represents the time range from 2004 to 2022 (Note: none of the retrieved literature was published in 2002 and 2003), and the red line represents the duration of the burst keywords.

In addition, keyword burst detection can also help us quickly identify research hotspots. Figure 9 shows 9 strongest citation bursts keywords for research related to bile acids and NAFLD between 2002 and 2022. The blue line represents the time range from 2004 to 2022 (Note: none of the retrieved literature was published in 2002 and 2003), and the red line represents the duration of the burst keywords. By the end of 2022, the nuclear receptor ranked first with the strength of 5.15, followed by FXR (4.1). Nuclear receptors and FXR have been found to play an important role in the treatment of NAFLD in recent years. Among all burst keywords, metabolic syndrome lasted the longest from 2013 to 2017, with the strength of 3.24. The strength of others was identification (3.86), HCC (3.61), progress (3.61), ligand (3.28), severity (3.12) and activation (3.09), respectively.

## 4 DISCUSSION

For this bibliometric analysis study, we searched the Web of Science database (SCI‐E) and screened 568 articles on bile acids and NAFLD from 2002 to 2022. The number of articles published generally showed a steady growth trend, especially since 2016, the number of publications in this field began to increase at a faster rate, so the research content has gradually become richer and enriched. In this study, the number and trend of articles published on bile acids and NAFLD were analysed from various aspects by using the online bibliometric analysis platform, CiteSpace and VOSviewer software. It showed the research viewpoints in this field in the past 20 years, and provided reference and guidance for future research. Therefore, most scholars can gain a general understanding of the relationship between bile acids and NAFLD through our bibliometric analysis, and quickly grasp the latest research hotspots in this field.

Over the past two decades, there has been a steady upwards trend in the number of annual articles published in the field of bile acids and NAFLD. It is worth noting that this growth rate has become faster since 2016, indicating that research on bile acids and NAFLD has become a global hotspot. From the Figure 3, it can be seen that the United States and China have made significant contributions to the field of bile acids and NAFLD. In the past two decades, the number of publications in the United States has maintained a steady growth. The research in this field in China started relatively late, and the number of publications for the first time in 2020 exceeded that in the United States. There is no doubt that the United States and China have played a leading role and made significant contributions in the study of bile acids and NAFLD. In addition, promoting cooperation between countries and regions has become an irreversible trend, and international cooperation models are more conducive to producing high‐quality research results. Of the countries/regions shown in Figure 4, the United States cooperates most closely with China, and these two countries are also the countries that participate most frequently in international cooperation. Among the 26 institutions in the top 10 in terms of the number of publications, 16 are from the United States, and their contributions to this field are mainly concentrated in recent years. In particular, the University of California in the United States has published the most articles (31), ranked first, as shown in the Table 2. Due to the late development of research in this field in China, the total number of publications in recent years has also been considerable. Therefore, these results demonstrate the in‐depth exploration and great potential for scientific innovation by American and Chinese scholars in the field of bile acids and NAFLD.

Among the top 10 most cited journals, *HEPATOLOGY* became the most cited journal (164) and its number of publications was the highest with a total of 22 articles. This journal is concerned with the physiology and pathology of hepatology, and NAFLD is an important component of liver disease research. In addition, the average number of citations per paper in the *LANCET* is the highest (89), which may be attributed to the high impact factor of the journal and the high academic quality of publications. Meanwhile, *LANCET* also had the highest impact factor (IF = 202.731) among the listed journals, followed by *GASTROENTEROLOGY* (IF = 33.883). Although *PLOS ONE* has a considerable number of citations of 67, its average number of citations per article is only 3.72, indicating that the articles published in it may have low reference value and thus fewer citations. Among the above listed 10 journals, 8 are from the United States, and the other journals were from the United Kingdom and the Netherlands. This fully demonstrates that the United States provides rich resources and platforms for research and development in the field of bile acids‐NAFLD.

CiteSpace was used to analyse and summarize the network of co‐cited references and keywords based on bibliographic records extracted from Web of Science,^24^ this can help us identify emerging trends in future research fields and potential hotspots in bile acids‐NAFLD related research. We first analysed the keywords in this field, so as to better grasp the frontier hotspots of the research. The top 10 keywords are listed as shown in Figure 8A, which are bile acids (213), nonalcoholic steatohepatitis (156), Farnesoid x receptor (137), fatty liver disease (121), expression (108), insulin resistance (107), metabolism (99), gut microbiota (88), inflammation (86) and mice (82). Based on the connection strength between keywords, we then performed cluster analysis on keywords and found that (Figure 8B) #1 cluster Obeticholic acid and #2cluster Oxidative stress were the larger groups among the 10 clusters. At present, the four main innovative methods for treating NAFLD include the use of anti‐oxidative stress drugs and the use of drugs targeting the FXR axis, namely obeticholic acid. It is a synthetic variant of the natural bile acid chenodeoxycholic acid, which acts as a ligand for FXR and may play a therapeutic role in NAFLD by activating FXR to reduce hepatic lipogenesis and degeneration.^25^ Relevant clinical studies have also confirmed that Obeticholic significantly improves fibrosis in NASH patients and key components of NASH disease progression.^26^ In addition, bile acids can be identified as biomarkers for non‐invasive diagnosis of NAFLD through metabolomic research methods.^27^ The activation of FXR reduces hepatic monounsaturated and polyunsaturated fatty acid (MUFA and PUFA) levels, which in turn reduces lipid accumulation by reducing bile acid absorption, this mechanism can effectively prevent NAFLD. FXR activation prevents NAFLD by reducing lipid absorption in bile acids dependent manner.^28^

Keywords burst refers to the high frequency of keywords cited by papers in a period of time, which can be used as an important indicator of research hotspots or emerging trends over time.^29^ As shown in Figure 9, we have listed 9 keywords with the highest burst strength, showing potential hotspots in bile acids‐NAFLD related research over the past two decades. It is worth noting that most of burst keywords listed in the figure began in 2013 and did not last until the end of 2022. This indicates that some of the research trends reflected by these keywords are showing an explosive state within a specific time frame, but some of their keywords cannot be considered as current hotspots in the field. Among them, the keyword metabolic syndrome, which lasted for the longest time from 2013 to the end of 2017, ranked first with a strength of 3.24. The research related to this keyword mainly focuses on the fields of insulin resistance, type 2 diabetes mellitus (T2DM) and NAFLD.^30^ The key factor of NAFLD is abnormal liver metabolism, and metabolic syndrome can be used as a more direct predictor of NAFLD. NAFLD is not only the cause but also the consequence of metabolic syndrome.^31^ There is a large number of evidence suggest that the relationship between NAFLD and metabolic syndrome is bidirectional, as patients with NAFLD may be predisposed to metabolic syndrome features, which in turn may exacerbate NAFLD or increase their unexpected risk of developing.^32^ Among the above burst keywords, nuclear receptor ranked the first with the strength of 5.15, followed by FXR with the strength of 4.1. As bile acids activated receptors, nuclear receptors and FXR constitute important components of bile acid signalling.^33^ The dysfunction of nuclear receptors which is a kind of transcription factors promotes the pathogenesis of NAFLD by affecting the regulation of energy metabolism by the liver‐adipose axis.^34^ Alterations in nuclear receptor signalling have often been found to be parallel to the metabolic disorders and participate in the pathogenesis of NAFLD. Nuclear receptors act as mediators linking the metabolic, inflammatory and fibrotic processes in NAFLD^35^

After analysing the top 10 articles with the highest number of citations in our research we found that the top 3 of them all described research related to the FXR signalling pathway. Interestingly this is consistent with the explosion of keywords burst and the time‐line of research in this field. In 2015 an article published by Neuschwander‐Tetri et al. from Saint Louis University in the Lancet journal ranked first in terms of citation.^18^ A multicentre randomized placebo‐controlled trial found that Obeticholic acid as a Farnesoid X nuclear receptor ligand improved the histological characteristics of NASH. The second article published in Gastroenterology described more about the efficacy and safety of the Farnesoid X receptor agonist obeticholic acid in patients with Type 2 diabetes and NAFLD.^19^ The third is an animal experiment related article published in the Journal of clinical investigation. The authors used a high‐fat diet‐induced NAFLD mouse model to determine the impact of gut microbiota alterations on NAFLD and found that inhibition of intestinal Farnesoid X receptor signalling was identified as a potential treatment for NAFLD.^20^ Similarly we analysed and visualized the co‐cited articles and found that more than half of them had studied FXR bile acids and gut microbiota at different levels including pathological physiological pharmacological and clinical studies. We found that the most co‐cited reference was published in HEPATOLOGY in 2017 which specifically discussed the relationship between bile acids and NAFLD as well as the possibility of treating NAFLD through bile acids related pathways.^21^ An animal experimental research article published in GUT in 2018 was also ranked first this study suggested that intervention in NAFLD can be achieved by targeting the FXR signalling pathway including bile acid conversion and gut microbiota.^22^ The two articles ranked second were published in HEPATOLOGY in 2018^23^ and in LANCET in 2015.^18^ These two articles respectively discuss the relationship between the presence and severity of NASH and specific changes in circulating bile acids as well as the improvement of the histological characteristics of NASH by Farnesoid X nuclear receptor ligand obeticholicacid. Furthermore, in addition to FXR as one of the receptors for bile acids we also cannot ignore the role of GPBAR1 (also known as TGR5). GPBAR1 was first discovered by researchers from the Takeda chemical industry to bind bile acids and induce cAMP in monocytes.^36^ Bile acids are the only known endogenous ligands for this receptor and in particular secondary bile acids have a higher affinity for it. Because GPBAR1 can bind conjugated bile acids it may act as an important regulator of bile acid homeostasis.^37^ The signalling pathway through activation of FXR and GPBAR1 these two main bile acid receptors play important roles in regulating glucose lipid and energy metabolism. For example agonists of FXR and GPBAR1 can improve glucose and insulin sensitivity and increase energy metabolism which can prevent obesity and NAFLD.^38^ GPBAR1 is highly expressed in liver sinusoidal epithelial cells Kupffer cells and the intestine which can convert ATP into cAMP and then activate cAMP‐related signalling pathways. Relevant clinical experiments have demonstrated that FXR and GPBAR1 signalling can improve hepatic bile acids metabolism and lipid homeostasis thereby preventing the development of NAFLD.^39^ FXR and GPBAR1 can also co‐ordinately regulate the signalling molecules related to metabolism and inflammation by binding with bile acids. These two important receptors mainly control the gene expression activity involved in inflammatory response by acting on enterohepatic tissue.^40^ GPBAR1 not only exerts anti‐inflammatory effects by inhibiting NF‐κB‐mediated production of pro‐inflammatory cytokines^41^ but also mediates effective anti‐inflammatory effects through activation of expression in monocytes and macrophages.^42^ The research and development of new drugs for these two important bile acids receptors is expected to become a new choice to improve glucose homeostasis regulate lipid metabolism and treat NAFLD‐associated liver injury.^43^ In the third place was an article on the analysis and evaluation of epidemiological related indicators of NAFLD published in HEPATOLOGY in 2016.^6^ Gut microbiota imbalance is a major feature of NAFLD and it can play a key role in the pathogenesis of NAFLD through its metabolites including changes in bacterial metabolites and interference with bile acids metabolism. Therefore the treatment of NAFLD by modulating intestinal microbiota is considerable.^44^ Current research indicates that gut microbiota also affects liver carbohydrate and lipid metabolism and influences the balance between pro‐inflammatory and anti‐inflammatory factors in the liver thereby affecting NAFLD and its progression to NASH.^45^ After reading the above articles we found that the publication time of the article and some keywords are consistent with the time‐Line chart in Figure 7 and keywords burst in Figure 9

In summary, obeticholic acid, bile acids, nuclear receptors, FXR, oxidative stress, gut microbiota and other factors are closely related to the occurrence and development of NAFLD, as well as its treatment methods. These factors reflect the hotspots and trends of future research and provide reference and guidance for subsequent research.

Our study may have certain limitations. Firstly, the data we analysed were extracted only from the SCI‐E database of WoSCC, excluding records from other major databases such as PubMed, Embase and Ovid. It results in the collected data are not fully representative of publications on bile acids and NAFLD over the past 20 years. However, the data retrieved from WoSCC included comprehensive records such as title, authors, institutions and references, which were necessary for bibliometric analysis. In addition, only the data retrieved from WoSCC rather than other databases include complete information such as references, so as to import into CiteSpace and VOSviewer software to complete citation analysis. Secondly, as English remains the preferred language for academic journals today, so our study only screened papers published in English, resulting in the omission of articles published in other languages.

## 5 CONCLUSION

In this study, CiteSpace, VOSviewer and bibliometric online analysis platform were used to analyse the related research fields of bile acids and NAFLD. Over the past 20 years, the number of publications in this field has shown a steady growth trend. Especially since 2013, the number of publications has increased at a relatively rapid rate, and the United States has maintained a relatively stable growth. China was late in the development of research in this field, and the number of publications began to increase at a high rate until 2020. The United States and China have made the greatest contributions to the research on bile acids and NAFLD, and have been in the leading position worldwide. At the same time, these two countries have the closest cooperation and exchange and actively participate in international cooperation. The current focus on bile acids, gut microbiota, and NAFLD plays an important role in further research. The intervention of NAFLD by gut microbiota via bile acids may be an important research direction in the future. This study provides a reference and guidance for further research on the mechanisms and treatment methods related to bile acids and NAFLD, and helps scholars better explore this field.

## AUTHOR CONTRIBUTIONS

**Cong Shibo:** Software (lead); visualization (lead); writing – original draft (lead); writing – review and editing (lead). **Wang Sili:** Resources (equal); writing – review and editing (equal). **Qiao Yanfang:** Writing – review and editing (equal). **Gu Shuxiao:** Writing – review and editing (equal). **Liu Susu:** Writing – review and editing (equal). **Chai Xinlou:** Project administration (equal). **Zhang Yongsheng:** Funding acquisition (lead); project administration (equal).

## FUNDING INFORMATION

The Fifth Batch of National Traditional Chinese Medicine Clinical Outstanding Talents Training Project (National Administration of Traditional Chinese Medicine talent education letter No. [2022] 1); The Sixth Batch of Academic Inheritance Training Program for Beijing Chinese Medicine Specialists (Beijing Chinese Medicine Section Word [2021] 169); The Third Zhongjing National Medical Seminar Training Program in Beijing (Beijing Chinese Medicine Science and Technology [2016] No. 114).

## CONFLICT OF INTEREST STATEMENT

The authors declare that they have no competing interests.

## Data Availability

All data generated or analysed during this study are included in this published article.
